# Sex Differences in Platelet Reactivity in Patients With ST-Elevation Myocardial Infarction: A Sub-Analysis of the ON-TIME 3 Trial

**DOI:** 10.3389/fcvm.2021.707814

**Published:** 2021-10-04

**Authors:** Anne H. Tavenier, Renicus S. Hermanides, Jan Paul Ottervanger, Svetlana V. Belitser, Olaf H. Klungel, Yolande Appelman, Maarten A.H. van Leeuwen, Arnoud W.J. van 't Hof

**Affiliations:** ^1^Department of Cardiology, Isala, Zwolle, Netherlands; ^2^Department of Pharmacoepidemiology, University of Utrecht, Utrecht, Netherlands; ^3^Department of Cardiology, Amsterdam UMC, Vrije Universiteit Amsterdam, Amsterdam, Netherlands; ^4^Department of Cardiology, Maastricht University Medical Centre, Maastricht, Netherlands; ^5^Department of Cardiology, Zuyderland Medical Centre, Heerlen, Netherlands

**Keywords:** ST-elevation myocardial infarction, platelet inhibition, ticagrelor, sex, gender

## Abstract

**Background:** Fast and adequate platelet inhibition is one of the cornerstones in the treatment of patients with ST-elevation myocardial infarction (STEMI). The aim of this analysis is to examine sex differences in platelet inhibition in the acute treatment of STEMI patients.

**Methods:** Platelet reactivity units (PRU) and ticagrelor plasma concentrations of all patients in the ON-TIME 3 were compared according to sex. All patients were pre-treated with crushed ticagrelor, aspirin and heparin. Both univariable and multivariable analyses were performed.

**Results:** In this sub-analysis of the ON-TIME 3 trial, 195 STEMI patients, of which 58 female patients (29.7%) and 137 male patients (70.3%), were analyzed. PRU-values immediately post-PCI were not different in females [median 135 (IQR 47-228)] compared to males [160 (IQR 40-219), *P* = 0.92]. Ticagrelor plasma concentrations were higher in the females at the start of primary PCI [141 ng/mL (IQR 25-491) vs. 76 ng/mL (IQR 15-245), *P* = 0.049] and at 6 hours post-primary PCI [495 ng/mL (IQR 283-661) vs. 321 ng/mL (IQR 196-537), *P* = 0.001] compared to males. However, immediately post-primary PCI and at 1-hour post-primary PCI no significant differences in ticagrelor concentrations were seen between sexes. In multivariable analysis, sex was significantly associated with ticagrelor concentration (*P* = 0.04), but not with PRU (*P* = 0.93).

**Conclusion:** Effective platelet inhibition reached by crushed ticagrelor in STEMI patients was similar in both sexes. Females had similar or even higher ticagrelor plasma concentrations up to 6 hours post-primary PCI compared with males.

## Introduction

The acute treatment of patients with ST-elevation myocardial infarction (STEMI) focuses on adequate antiplatelet therapy and timely revascularization of the culprit vessel by a primary percutaneous coronary intervention (PCI) (1, 2). Fast and adequate platelet inhibition is often reached by (pre-hospital) administration of intravenous (iv) aspirin and a potent P2Y_12_ receptor inhibitor, such as ticagrelor and prasugrel. The European STEMI guideline highlights that females and males receive equal benefit from reperfusion therapy and other STEMI-related therapies (2). Although sex differences in cardiology are of increasing interest in research, sex differences in platelet inhibition in the acute treatment of STEMI patients are relatively undetermined. Some studies show increased platelet reactivity in healthy females or female patients undergoing elective PCI compared to their male counterparts (3–5), while other studies did not find such an effect in patients with an acute coronary syndrome (ACS) or elective PCI (6–8). In healthy individuals, females had higher ticagrelor concentrations than males after a single high dose ticagrelor (9). A similar efficacy and safety profile of ticagrelor has been described in females and males with an ACS (10). Studies regarding sex differences in pharmacodynamics and -kinetics of ticagrelor in the acute phase of STEMI are scarce.

In this sub-analysis of the ON-TIME 3 trial we examine sex differences in platelet inhibition and ticagrelor plasma concentrations in the acute phase of STEMI.

## Methods

### Study Design and Patients

The ON-TIME 3 trial was an investigator-initiated, randomized, open-label, multicenter study that randomized STEMI patients, who were pre-treated with aspirin and crushed ticagrelor, to fentanyl or acetaminophen iv in a pre-hospital setting. The main results showed higher absorption of ticagrelor with acetaminophen iv compared fentanyl without a significant difference in platelet inhibition and pain relief. The results have been published previously (11). The study was approved by the ethics committee of Zwolle (the Netherlands) and was conducted in accordance with the principles of the Declaration of Helsinki.

The inclusion and exclusion criteria have been published before (12). In brief, patients with signs or symptoms of STEMI and a pain score of 4 or higher on a 10-step numeric rating pain score who were planned to undergo a primary PCI and who were P2Y_12_ naive, were enrolled. This sub-analysis focuses on sex differences in platelet reactivity and ticagrelor concentrations in these patients.

### Study Procedures

Pre-hospital treatment usually included a loading dose of aspirin (Aspegic 500mg IV), a loading dose of crushed ticagrelor 180 mg, and 5,000 IU of heparin. All patients were randomized to acetaminophen iv (1,000 mg) or fentanyl iv (1–2 mcg/kg). As only a minority of our patients underwent angiography only, we will refer to the time points with regard to PCI in this article. Data on platelet inhibition, including pharmacokinetics and pharmacodynamics, were collected before (T1) and immediately after primary PCI (T2), and at 1-hour post-primary PCI (T3) and 6 hours post-primary PCI (T4).

Pharmacodynamics were assessed by a VerifyNow P2Y_12_ point of care test (Accriva, San Diego, CA) for measurement of platelet reactivity units (PRU). Pharmacokinetics were evaluated by determination of the concentration of ticagrelor and its active metabolite, AR-C124910XX, using liquid chromatography-mass spectrometry in the clinical chemistry laboratory in Zwolle.

### Study Endpoints

The primary endpoint of the study was the level of platelet reactivity units (PRU) measured immediately post-primary PCI (T2). For the assessment of the primary endpoint, blood was obtained just before sheath removal in case of a primary PCI. Secondary endpoints included the level of PRU at other time points, high on platelet reactivity (HPR) defined as PRU >208 (13) measured immediately post-primary PCI, the plasma concentrations of ticagrelor, its active metabolite and the cumulative plasma concentrations of ticagrelor and its active metabolite at all time points. Exploratory endpoints included major adverse cardiac events, including reinfarction, target vessel revascularization, stent thrombosis, death and BARC 3 and 5 bleeding (14), and all bleeding (BARC 1–5).

### Statistical Analysis

Patients were analyzed as females vs. males. Continuous variables were compared using Student's *t*-test and presented as mean and standard deviation (SD), or as median and interquartile range (IQR) and compared with Mann Whitney U test if they were non-normally distributed. Categorical variables are presented as numbers and percentages and compared using Pearson's chi square test or Fisher exact test. Univariable and multivariable analyses were performed for all endpoints. Additionally, a sensitivity analysis using multiple imputation for missing values was performed. Multivariate linear mixed effect modeling did not fulfill its assumptions. Therefore, we used non-linear quantile regression techniques for modeling of our data. Potential confounders included in our analyses were age, study medication (IV acetaminophen or IV fentanyl), hypertension, renal function, platelet count and BMI. In this analysis the exact time after randomization was used with time on a continuous scale. Bootstrapping was used to determine the median differences and their confidence intervals in PRU or ticagrelor concentrations between both sexes at multiple timepoints. A *p*-value below 0.05 was considered statistically significant. All analyses were performed with R version 3.6.0.

## Results

### Patient Characteristics

All 195 patients included in the ON-TIME 3 study, were included in the current analysis, of which 58 female patients (29.7%) and 137 male patients (70.3%). Baseline, angiographic and electrocardiographic characteristics are shown in Table 1. The two groups differed on a few baseline characteristics like age (68.2 years in females vs. 61.9 years in males, *P* < 0.001), hypertension (53.4 vs. 33.6%, *P* = 0.01), and renal function based on creatinine levels (69 (IQR 62–81) vs. 87 (IQR 76–97), *P* < 0.001).

**Table 1:** Baseline, angiographic and electrocardiographic characteristics.

	**Female patients** ***N* = 58**	**Male patients** ***N* = 137**	***P*-value**
**General baseline characteristics**			
Age (mean, SD) (%)	68.2 (9.8)	61.9 (11.4)	<0.001
Fentanyl arm	33 (56.9)	64 (46.7)	0.25
Diabetes mellitus (%)	13 (22.4)	21 (15.3)	0.32
Hypertension (%)	31 (53.4)	46 (33.6)	0.01
Hypercholesterolemia (%)	18 (31.0)	39 (28.5)	0.85
Smoking			0.45
Non-smoker (%) In the past (%) Current (%)	24 (43.6) 10 (18.2) 21 (38.2)	45 (34.1) 26 (19.7) 61 (46.2)	
Family history of CAD (%)	22 (37.9)	70 (51.1)	0.33
Peripheral artery disease (%)	1 (1.7)	3 (2.2)	1.00
Prior myocardial infarction (%)	4 (6.9)	15 (10.9)	0.54
Prior PCI (%)	3 (5.2)	19 (13.9)	0.13
Prior CABG (%)	0 (0)	1 (0.7)	1.00
BMI (median, IQR)	25.4 [22.7–30.8]	24.4 [29.6]	0.21
Platelet count (median, IQR)	269 [235–319]	242 [208–285]	0.02
Renal function based on creatinine (median, IQR)	69 [62–81]	87 [76–97]	<0.001
CK max. (U/L; median, IQR)	661 [266–1220]	893 [387–2025]	0.71
CK MB max. (U/L; median, IQR)	84 [37–157]	102 [49–228]	0.35
Troponine T max. (ng/mL; median, IQR)	1.350 [0.466–3.300]	2.200 [0.668–5.275]	0.38
Killip class I (%)	54 (93.9)	135 (98.5)	0.07
Vomiting (%)	4 (6.9)	13 (9.5)	0.76
Level of pain on 10-step pain scale at randomization (median, IQR)	8 (6–8)	7 (6–8)	0.23
Time from symptom onset to T1 in mins (median, IQR)	138 [92–193]	133 [90–226]	0.98
Time from randomization to T1 in mins (median, IQR)	64 [52–79]	65 [52–78]	0.91
Time from randomization to T2 in mins (median, IQR)	101 [82–123]	100 [84–118]	0.89
Time from randomization to T3 in mins (median, IQR)	179 [158–203]	181 [151–202]	0.90
Time from randomization to T4 in mins (median, IQR)	488 [460–515]	487 [453–518]	0.81
**Angiographic characteristics**			
Radial access site (%)	56 (96.6)	126 (92.0)	0.35
Type of procedure			0.85
CAG only (%) POBA only (%) Primary PCI (%)	6 (10.3) 4 (6.9) 48 (82.8)	13 (9.5) 7 (5.1) 117 (85.4)	
Culprit			0.51
LAD (%) RCA (%) RCx (%) LM (%) Arterial graft (%) Venous graft (%) Other/no culprit (%)	17 (29.3) 31 (53.4) 8 (13.8) 1 (1.7) 0 (0) 0 (0) 1 (1.7)	47 (34.3) 68 (49.6) 13 (9.5) 1 (0.7) 0 (0) 0 (0) 8 (5.8)	
Thrombus aspiration (%)	11 (19.0)	29 (21.2)	0.88
TIMI flow grade pre-procedure (%)			0.95
0 1 2 3	26 (50.0) 6 (11.5) 10 (19.2) 10 (19.2)	67 (54.0) 12 (9.7) 21 (16.9) 24 (19.4)	
TIMI flow grade post-procedure (%)			0.55
0 1 2 3	1 (1.7) 0 (0) 1 (1.7) 56 (96.6)	1 (0.7) 0 (0) 5 (3.6) 131 (95.6)	
Myocardial blush grade (%)			0.92
Unknown 0 1 2 3	3 (5.2) 1 (1.7) 2 (3.4) 9 (15.5) 43 (74.1)	10 (7.3) 4 (2.9) 4 (2.9) 26 (19.0) 93 (67.9)	
Distal embolization (%)	4 (6.9)	7 (5.1)	0.74
Glycoprotein IIb/IIIa inhibitor (%)			0.20
None 6 hours infusion 12 hours infusion 24 hours infusion	48 (82.8) 4 (6.9) 5 (8.6) 1 (1.7)	111 (81.0) 19 (13.9) 4 (2.9) 3 (2.2)	
**Electrocardiographic results**			
Complete ST-resolution (%)			
Immediately after primary PCI 1 hour after primary PCI 6 hours after primary PCI	26 (51) 31 (63.3) 40 (75.5)	36 (31.6) 58 (46.4) 80 (67.2)	0.08 0.23 0.65

*BMI, body mass index; CAD, coronary artery disease; CABG, coronary artery bypass grafting; CK, creatine kinase; CK MB, creatine kinase myoglobin binding; IQR, interquartile range; PCI, percutaneous coronary intervention; SD, standard deviation, T1, before primary PCI; T2, immediately after primary PCI; T3, 1-hour after primary PCI; T4, 6 hours after primary PCI; CAG, coronary angiography; LAD, left anterior descending artery; LM, left main vessel; POBA, plain old balloon angiography; PCI, percutaneous coronary intervention; RCA, right coronary artery; RCx, ramus circumflex artery; TIMI, Thrombolysis In Myocardial Infarction; PCI, percutaneous coronary intervention*.

Female patients experienced similar levels of pain at randomization compared to males (8 out of 10 step pain scale (IQR 6–8) for females; 7 out of 10 step pain scale (IQR 6–8) for males; *P* = 0.23).

The median times from arrival of the ambulance at the patient site to arrival at the cathlab (64 minutes (52–79) vs. 65 minutes (52–78), *P* = 0.91) and end of the primary PCI (103 minutes (82–116) vs. 100 minutes (84–118), *P* = 0.72) were similar in females compared to males, respectively.

### Pharmacodynamics

**Figure 1:**
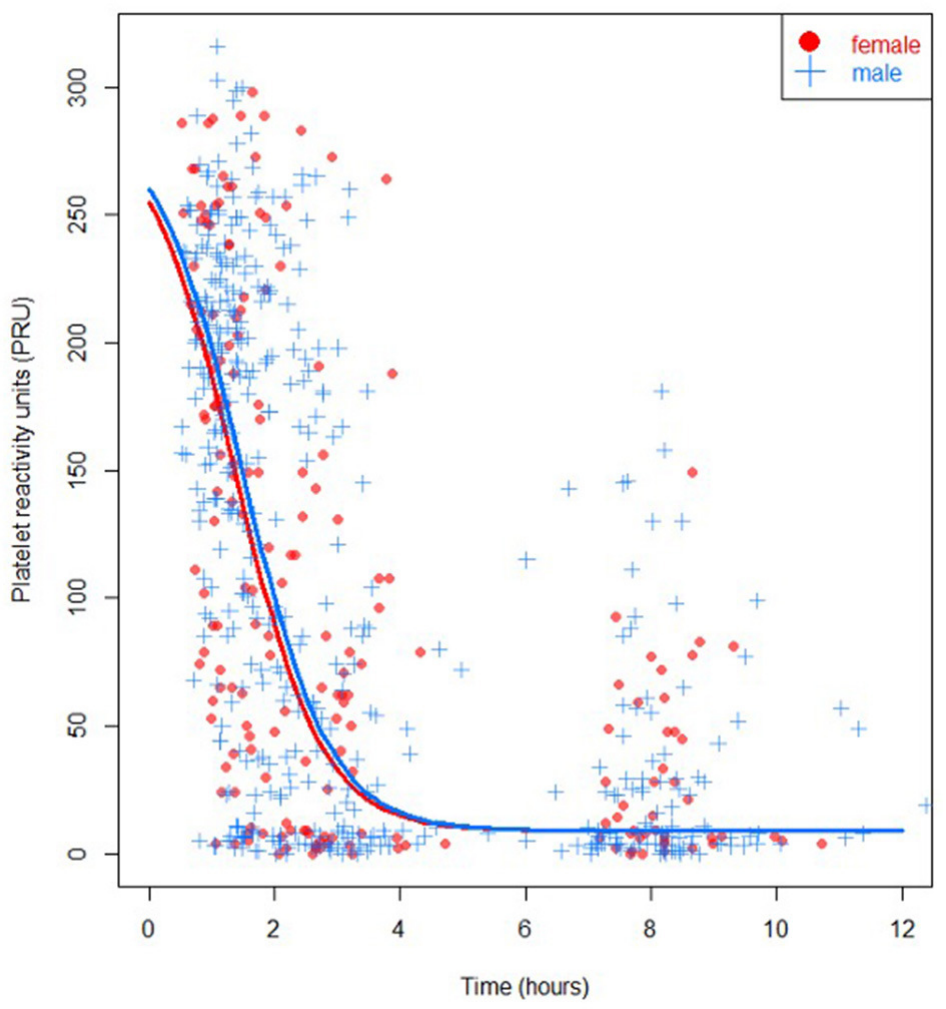
PRU specified for sex (*P* = 0.93), females in red, males in blue.

PRU-values were available in 83% of measurements in females and in 82% of measurements in males. Reasons for missing values were measurement or logistical errors. In univariable analysis, the PRU-value immediately post-PCI (or 1 hour after angiography), was not different in females compared to males (median 135 (IQR 47–228) and 160 (IQR 40–219) respectively, *P* = 0.92). Also, pre-primary PCI and at 1 hour and 6 hours post-primary PCI no statistically significant differences in PRU between females and males were found (Table 2, Figure 1). Moreover, no statistically significant differences in high platelet reactivity (HPR) measured immediately post-primary PCI were seen between females and males (34.0 vs. 30.7%, *P* = 0.81). Similar results were seen in a sensitivity analysis using multiple imputation (Supplementary Material).

**Table 2:** Primary and secondary outcomes on pharmacodynamics and–kinetics.

**Main outcomes**	**Female patients** ***N* = 58**	**Male patients** ***N* = 137**	*P*-value^*^
PRU (median, IQR)			
T1 T2 T3 T4	174 [86–227] 135 [47–228] 45 [6–99] 13 [6–48]	188 [122–224] 160 [40–219] 40 [7–127] 8 [4–33]	0.75 0.92 0.60 0.33
High platelet reactivity at T2 (%)	17 (34.0)	35 (30.7)	0.81
Ticagrelor concentration (median, IQR)			
T1 T2 T3 T4	141 [25–491] 270 [74–799] 474 [238–904] 495 [283–661]	76 [15–245] 163 [37–414] 408 [179–708] 321 [196–537]	0.049 0.09 0.13 0.001
Ticagrelor active metabolite concentration (median, IQR)			
T1 T2 T3 T4	12 [0–47] 34 [7–117] 93 [35–212] 160 [96–206]	4 [0–22] 17 [0–62] 81 [22–131] 92 [54–134]	0.04 0.049 0.09 <0.001
Ticagrelor concentration total (median, IQR)			
T1 T2 T3 T4	150 [25–555] 205 [88–945] 556 [272–1099] 656 [395–850]	76 [16–271] 188 [43–468] 485 [201–823] 431 [264–632]	0.05 0.09 0.12 <0.001

* *univariable analysis*.

*IQR, interquartile range; PRU, platelet reactivity units; T1, before primary PCI; T2, immediately after primary PCI; T3, 1-hour after primary PCI; T4, 6 hours after primary PCI*.

In multivariable analysis, sex was not significantly associated with PRU-value (*P* = 0.93). At 1 hour after randomization, corresponding with measurements performed pre-PCI (T1), and at 100 minutes after randomization, corresponding with measurements performed immediately after primary PCI (T2), the median PRU of females did not differ from the PRU of males (median difference −11 (IQR −36–14) and −19 (IQR −62–28) respectively, using bootstrapping). Also, at 3 hours post-randomization, corresponding to measurements performed 1 hour after PCI (T3), the median PRU did not significantly differ between sexes (−5 (IQR −31–23)). No reliable multivariable result could be presented due to instability of the model at 8 hours post-randomization (corresponding to 6 hours after PCI; T4). Higher platelet counts were associated with lower PRU-values in the multivariable model (*P* = 0.01).

### Pharmacokinetics

**Figure 2:**
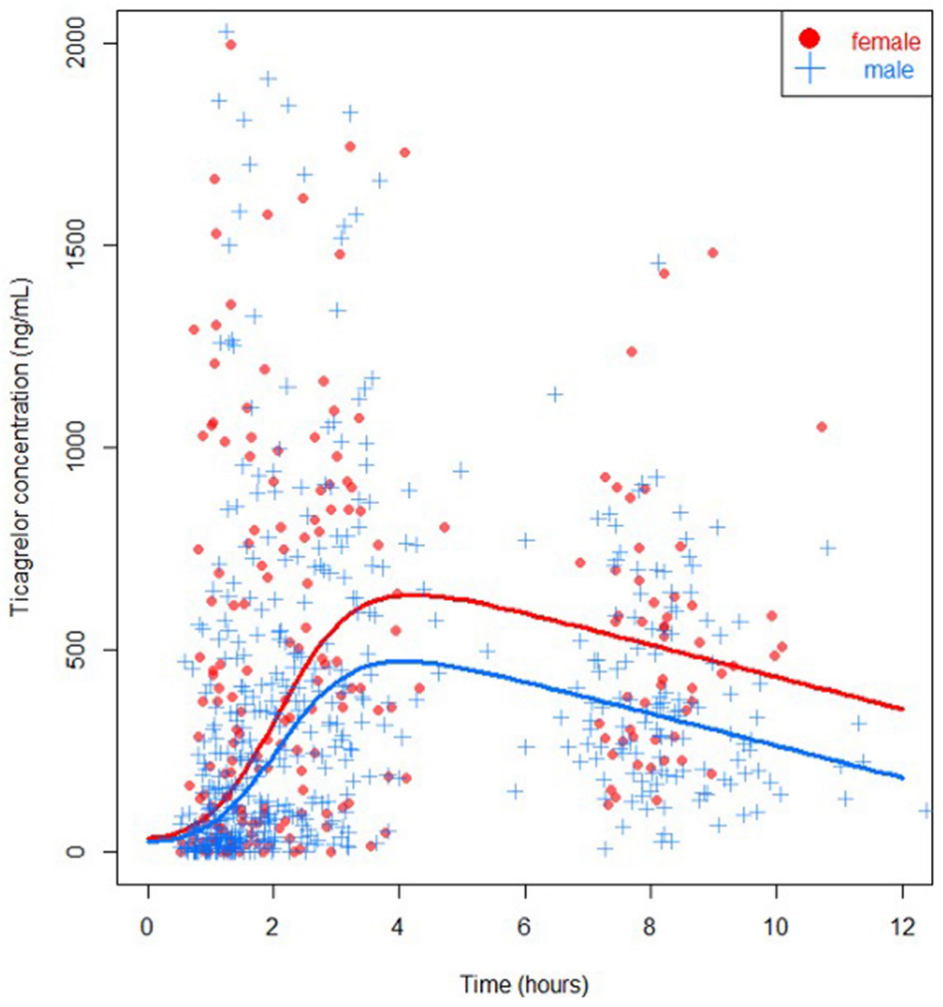
Ticagrelor concentration specified for sex (*P* = 0.04), females in red, males in blue.

Ticagrelor values were available in 94% of measurements in females and in 95% of measurements in males. Missing values were due to logistical errors. In univariable analysis, ticagrelor concentrations were higher in females at the start of primary PCI (141 ng/mL (IQR 25–491) vs. 76 ng/mL (IQR 15–245), *P* = 0.049) and at 6 hours post-primary PCI (495 ng/mL (IQR 283–661) vs. 321 ng/mL (IQR 196–537), *P* = 0.001) (Table 2, Figure 2). However, immediately and at 1-hour post-primary PCI no statistically significant differences were found between females and males (270 ng/mL (IQR 74-799) vs. 163 ng/mL (IQR 37–414), *P* = 0.09; and 474 ng/mL (IQR 238–904) vs. 408 ng/mL (IQR 179–708), *P* = 0.13; respectively). Similar results were seen for the cumulative concentration of ticagrelor and in the sensitivity analysis using multiple imputation (Supplementary Material). The active metabolite of ticagrelor measurements differed in favor of females on all timepoints except at 1-hour post-primary PCI.

In multivariable analysis, female sex was significantly associated with higher levels of ticagrelor concentration (*P* = 0.04). BMI did not modify this association significantly (*P* = 0.45), but a higher platelet count was also associated with a higher ticagrelor concentration in the multivariable model (*P* = 0.02). When focusing specifically on each time point using bootstrapping, female sex was non-significantly associated with higher levels of ticagrelor concentration at 1-hour after randomization [corresponding to measurements performed pre-PCI (T1); median difference 57 ng/mL (IQR −6–195)], at 100 minutes after randomization [corresponding to measurements performed immediately after primary PCI (T2); median difference 97 ng/mL (IQR −17–222)], at 3 hours after randomization [corresponding to measurements performed 1 hour after primary PCI (T3); median difference 102 ng/mL (IQR −37–237)], and at 8 hours after randomization [corresponding to measurements performed about 6 hours after PCI (T4); median difference 132 (IQR −61–291)].

### Clinical Endpoints

Within 30 days after STEMI three re-infarctions and two BARC 3 bleedings occurred in females. In males one acute stent thrombosis was seen. When taking into account all bleeding events, females had additionally 7 (12.1%) BARC 1 bleeding events and males had 11 (8%) BARC 1 and 6 (4.4%) BARC 2 bleedings events. Bleeding events were slightly more frequent in females (overall difference in bleeding events 15.5 vs. 12.4%, *P* = 0.048).

## Discussion

This sub-analysis of the ON-TIME 3 trial showed that effective platelet inhibition with crushed ticagrelor was reached at multiple time points prior and after primary PCI in both female and male patients with STEMI. However, ticagrelor plasma concentrations were higher in females compared with males at several timepoints.

Literature shows higher platelet reactivity in healthy females, compared to males, who were not treated with antiplatelet therapy (3, 4). Moreover, higher platelet reactivity was measured in female patients, who underwent elective PCI and were treated with aspirin and clopidogrel compared to their male counterparts (5). However, in another study among patients treated with dual antiplatelet therapy, no effect of sex was seen on platelet reactivity or high-on treatment platelet reactivity, a phenomenon associated with major cardiovascular events (15), at 30–90 days after an acute coronary syndrome (ACS) or elective PCI (6). Furthermore, sex was not a significant predictor of high-on platelet reactivity in post-ACS patients treated with aspirin and prasugrel for 1–3 months after discharge (7). Also, a sub-analysis of the ADAPT-DES study showed that the association of HPR and stent thrombosis at 1 year was similar in females and males (8).

Abovementioned studies on platelet reactivity were performed in the sub-acute or chronic phase after initiation of dual antiplatelet therapy for ACS or elective PCI and showed inconclusive results. Importantly, our analysis focused on sex differences in P2Y_12_ platelet inhibition in the acute phase of STEMI and did not show any significant differences.

In this sub-analysis ticagrelor absorption was higher in females compared with males at several timepoints, which could potentially have resulted in slightly more observed overall bleedings in females. Suggested explanations may include accompanied differences in body fat distribution and body mass index (BMI) between females and males. Though, in this analysis BMI did not significantly modify the association of sex and ticagrelor concentration. Also, current use of P2Y_12_ inhibitors was an exclusion criterion and, therefore, cannot explain the differences in ticagrelor concentrations found between sexes based on cardiovascular history. Moreover, no significant differences in infarct size, based on maximum cardiac markers, were found between sexes and discourage a hypothesis of lower intestinal absorption of ticagrelor in men due to hemodynamic changes as a result of larger infarctions. Similar sex differences in ticagrelor concentration as in our study were found in healthy individuals after a single loading dose of ticagrelor (9). The mechanism behind these findings is not fully clarified yet, but one theory is that the activity of P-glycoprotein, related to elimination of compounds of hepatocytes and enterocytes (16, 17), is potentially lower in females (18). Although these sex differences on P-glycoprotein activity are not evident, lower metabolic activity could result in lower biliary excretion of ticagrelor and its active metabolite and therefore increasing its plasma levels (9, 16).

Additionally, while knowing that most excretion of ticagrelor occurs via feces and to a less extent via urine (16), renal function did not modify our results significantly.

Females have a greater expression of CYP3A4 (19), an enzyme involved in the metabolization of ticagrelor (20), and this might have influenced ticagrelor plasma concentrations in our study. In a genome-wide association study (GWAS), plasma levels of ticagrelor were associated with two single nucleotide polymorphisms (SNPs) in the CYP3A4 region (21). However, the effects on plasma concentrations of each of these loci were small and did not result in differences on clinical outcome.

Furthermore, in this study ticagrelor concentrations were higher in females at several timepoints in the acute phase of STEMI but the use of glycoprotein IIb/IIIa inhibitors (GPI) was comparable in both sexes. Since GPI increases risk of the bleeding, and female gender is associated with bleeding (22), a suggestion may be to be more restrictive to administer GPI in females as their ticagrelor absorption profile is more beneficial and no or less additional platelet inhibition is required.

High maintenance dose of aspirin has been suggested to outweigh the platelet inhibitory effects of ticagrelor, but not of clopidogrel, by inhibiting prostaglandin (23, 24). Moreover, differences were described in efficacy of aspirin between females and males (3), and this raises a question about the presence of sex differences in the aspirin-ticagrelor interaction. However, in our study we were not able to analyze such effects because all patients received 80 mg of aspirin (low dose) and no specific measurements for aspirin platelet response were performed.

Furthermore, the influence of female hormones on platelet inhibition in STEMI is also not fully clarified yet. Endogenous estrogens are possibly related to no-reflow phenomenon in STEMI in postmenopausal females (25), but further research is required to assess the influence of estrogens on P2Y_12_ platelet inhibition in STEMI.

The platelet counts slightly differed between sexes in our cohort. Studies showed that higher platelet counts may result in higher risk of thrombosis by providing a higher substrate for platelet-fibrin thrombus formation (26, 27). Moreover, platelet count may be used as a marker for systemic inflammation and source of inflammatory mediators (26). A lower platelet count, thrombocytopenia, has been associated with a higher bleeding risk (26) and has been associated with worse mortality outcome in patients with acute coronary syndrome (28, 29). High or low platelet count as a risk factor for adverse outcome has also been illustrated by a recently developed prediction model of cardiac arrest (30). In our study, platelet count was a significant variable in the multivariable models for PRU and ticagrelor concentrations but did not change our main conclusions regarding sex differences. Moreover, higher platelet counts were associated with lower PRU-values in the multivariable model, which was contradictive with the abovementioned studies.

### Impact of P2Y_12_ Inhibition on Clinical Outcome Sex-Specified

A higher mortality was found in young females (<65 years) compared to young males treated with primary PCI even after correction of time delay before primary PCI (31–34). Moreover, a sub-analysis of the ATLANTIC trial, which randomized STEMI patients to pre-hospital or in-hospital ticagrelor, observed a small increase in short-term all-cause mortality in females compared with males (35). Apart from other mechanisms, these results could potentially be caused by sex differences in platelet inhibition. In this study, however, no sex differences on P2Y_12_ platelet inhibition were found, implying also no translation to differences on clinical outcomes between females and males based on P2Y_12_ platelet inhibition. Illustratively, a sub-analysis of the PLATO trial, which randomized ACS patients to ticagrelor or clopidogrel within 24 hours of symptoms and before PCI, showed that female sex was not an independent risk factor for adverse clinical outcomes and that ticagrelor has a similar efficacy and safety profile in females and males (10). Moreover, in a large meta-analysis of randomized trials of potent P2Y_12_ inhibitors the efficacy and safety of potent P2Y_12_ inhibitors were comparable between females and males (36), suggesting no patient selection for P2Y_12_ inhibition based on sex. A sub-analysis of the CHAMPION-PHOENIX trial, which randomized patients undergoing elective PCI or urgent PCI to cangrelor or clopidogrel, also found consistent beneficial effects of cangrelor in both sexes. Only a small increase in moderate bleeding was observed in cangrelor treated females, but not in cangrelor treated males (37). In our study, we observed slightly more bleeding events in females than males. However, our sample size was too small to analyze such an effect on clinically relevant bleeding.

Some limitations need to be acknowledged. This sub-analysis was not pre-specified, and the study was therefore not designed to primarily assess sex differences. However, since all patients received similar therapy and sex is specified before STEMI presentation, confounding by indication or other forms of selection bias were less likely present. The number of females in our study was too small to detect differences on clinical outcomes and possibly lack power to find differences in platelet inhibition. Differences in baseline characteristics were corrected for, such as age, given study medication (acetaminophen or fentanyl), hypertension, renal function and BMI, but probably there are factors that could not be adjusted for.

In this study, sex was significantly associated with levels of ticagrelor concentration but not with PRU. This discrepancy might be due to more missing values of PRU (82% available) compared to ticagrelor concentration levels (94% available) with resulting consequences in power. However, a sensitivity analysis with multiple imputation did not show a significant association between sex and PRU-values either. Also, aspirin induced platelet reactivity was not studied in this analysis. Furthermore, this study focused on the acute phase of STEMI but did not study the long-term effects of platelet inhibition and sex. Future research may focus on potential sex differences on long-term effects of platelet inhibition in the acute phase of STEMI and their translation to clinical events.

## Conclusion

Effective platelet inhibition is reached by pretreatment with crushed ticagrelor in the acute phase of STEMI in both sexes. Female patients had similar or even higher ticagrelor plasma concentrations up to 6 hours post-primary PCI compared with male patients.

## Data Availability Statement

The original contributions presented in the study are included in the article/Supplementary Material, further inquiries can be directed to the corresponding author/s.

## Ethics Statement

The ON-TIME 3 trial was reviewed and approved by the METC Isala Zwolle. The patients provided their verbal and written informed consent to participate in this study.

## Author Contributions

AT, RH, SB, and AH: methodology. AT and SB: formal analysis. AT: data curation. AT: writing—original draft preparation. AT, RH, JO, SB, OK, YA, ML, and AH: writing—review & editing. AH: supervision. All authors contributed to the article and approved the submitted version.

## Funding

The ON-TIME 3 trial was conducted with an unrestricted grant from AstraZeneca. However, AstraZeneca was not involved in the analysis and writing of this sub-analysis.

## Conflict of Interest

ML reports a research grant from AstraZeneca (REDUCE-MVI trial) and consultant fees from Terumo corp. AH reports institutional fees and non-financial support from AstraZeneca as well as grants from Medtronic. The remaining authors declare that the research was conducted in the absence of any commercial or financial relationships that could be construed as a potential conflict of interest.

## Publisher's Note

All claims expressed in this article are solely those of the authors and do not necessarily represent those of their affiliated organizations, or those of the publisher, the editors and the reviewers. Any product that may be evaluated in this article, or claim that may be made by its manufacturer, is not guaranteed or endorsed by the publisher.
