# Supplementary material for: Sex Differences in Platelet Reactivity in Patients With ST-Elevation Myocardial Infarction: A Sub-Analysis of the ON-TIME 3 Trial
Source: Front Cardiovasc Med. 2021 Oct 4;8:707814. doi: 10.3389/fcvm.2021.707814 (PMC8520931; doi:10.3389/fcvm.2021.707814)
Supplement: Supplementary file 1 [file Table_1.docx]

**Supplementary material**

**Sensitivity analysis**

- Using multiple imputation:

| **Main outcomes with imputation** | **Female patients**  **N = 58** | **Male patients**  **N = 137** | **P-value** |
| --- | --- | --- | --- |
| PRU (mean, SE)  T1  T2  T3  T4 | 157 (10.8)  130 (13.4)  60 (13.1)  29 (5.9) | 164 (6.6)  143 (8.3)  74 (8.3)  29 (3.7) | 0.58  0.40  0.36  0.96 |
| High platelet reactivity op T2 (%) | 18 (31.0) | 42 (30.5) | 0.94 |
| Ticagrelor concentration (mean, SE)  T1  T2  T3  T4 | 367 (61.1)  472 (70.4)  633 (68.6)  555 (54.8) | 243 (34.8)  358 (42.4)  498 (33.3)  386 (20.8) | 0.06  0.15  0.046  <0.001 |
| Ticagrelor active metabolite concentration (mean, SE)  T1  T2  T3  T4 | 37 (6.8)  76 (11.9)  140 (17.9)  168 (14.4) | 31 (5.4)  55 (7.2)  105 (10.8)  101 (5.1) | 0.49  0.12  0.09  <0.001 |
| Ticagrelor concentration total (mean, SE)  T1  T2  T3  T4 | 404 (67.2)  548 (80.8)  773 (84.7)  724 (66.4) | 274 (39.0)  412 (48.4)  603 (41.1)  487 (24.4) | 0.08  0.14  0.04  <0.001 |

PRU: platelet reactivity units; SE: standard error; T1: before primary PCI; T2: immediately after primary PCI; T3: 1-hour after primary PCI; T4: 6 hours after primary PCI
